# Quantification of amino acids in small volumes of palm sweat samples

**DOI:** 10.1016/j.heliyon.2024.e36286

**Published:** 2024-08-20

**Authors:** Makoto Tsunoda, Takao Tsuda

**Affiliations:** a Graduate School of Pharmaceutical Sciences, University of Tokyo, Tokyo, Japan; b Pico-device, Nagoya, Japan

**Keywords:** Diagnosis, Liquid chromatography, Fluorescence

## Abstract

This study investigates the significance of quantifying amino acids in minute sweat volumes using high-performance liquid chromatography with fluorescence detection. Sweat, a valuable biofluid for non-invasive health monitoring, provides real-time insights into physiological changes. Amino acids, which are critical for various physiological processes, are key to protein synthesis and cellular regulation. Therefore, analyzing sweat's amino acid profiles can offer insights into metabolic states, exercise-induced stress, and potential biomarkers for health conditions. For sensitive analysis, amino acids were derivatized with 4-fluoro-7-nitro-2,1,3-benzoxadiazole (NBD-F), followed by liquid chromatographic separation on an octadecylsilyl column and fluorescence detection. The developed method was validated and applied to human sweat samples, enabling the quantification of 14 amino acids. The most abundant amino acids in the samples were serine, glycine, and alanine, which aligns with prior studies. This method offers a non-invasive and efficient way to screen for diseases by detecting amino acids in sweat, even with minimal sweat volumes. The approach could also be used to analyze other biomolecules in sweat, expanding its potential applications.

## 1 Introduction

The measurement of metabolites in biological samples other than blood has gained increasing significance due to the rising demand for non-invasive analytical methods. Sweat, saliva, and tears have emerged as particularly relevant sources for such analyses [1]. Of these, sweat is notable as a biofluid that allows for non-invasive, continuous, and real-time health monitoring [[2], [3], [4], [5]].

Sweat is primarily produced by sweat glands to regulate body temperature, hydrate the skin, and provide protection. Comprising about 99 % water, sweat also contains essential ions like chloride, sodium, and potassium, along with smaller amounts of calcium, magnesium, lactate, and other minor electrolytes [6,7]. Sweat also contains trace amounts of various substances that can offer valuable insights into a person's health status. Analyzing sweat has become crucial for non-invasive health monitoring. Continuous monitoring of sweat metabolites can offer real-time information on changes in the body's physiological and metabolic state, making it a promising approach for personalized medicine and disease management [8,9]. The non-invasive nature of sweat collection is especially advantageous, providing a more comfortable and convenient alternative to traditional blood sampling. Sweat testing is a diagnostic procedure used to analyze the composition of sweat, and it's particularly useful in the medical field. It's most commonly used to diagnose cystic fibrosis (CF), a genetic disorder that affects the respiratory system, digestive system, and sweat glands [[10], [11], [12]]. The high levels of chloride in the sweat of CF patients are a key diagnostic marker, demonstrating the potential of sweat analysis for diagnosing certain medical conditions.

Amino acids are fundamental to a variety of physiological processes in the body. They serve as the building blocks of proteins, influence protein turnover and cellular biology, and participate in regulation and transport mechanisms. Determining amino acid concentrations is significant in many fields, such as biological research, clinical diagnosis, and food chemistry. The concentration of free amino acids in biological samples like blood, urine, and cerebrospinal fluid provides critical biochemical indicators [13,14]. These measurements are key to identifying metabolic disorders in newborns and monitoring the effectiveness of therapeutic interventions. Accurate analysis of amino acids in these samples offers valuable insights into metabolic pathways, enabling early diagnosis and effective management of various conditions. Furthermore, amino acid profiling enhances our understanding of nutritional status and metabolic health in both research and clinical settings [15,16]. Analyzing the amino acid composition of sweat can provide insights into an individual's metabolic state, exercise-induced stress, and potential biomarkers for various health conditions [[17], [18], [19], [20]]. Several studies have examined amino acid profiles in sweat to understand how they change under different physiological conditions. For example, one study found that the amino acid content in sweat decreased over the course of exercise [21]. These shifts in amino acid concentrations can reflect the intensity and duration of physical activity.

The most common sweat testing method involves collecting perspiration from a specific area of the skin, typically the forearm. This process uses iontophoresis, a technique where a substance that stimulates sweat gland is applied to the skin with the help of a precise electric current [22]. Participants undergoing this procedure often report no discomfort or burning sensations. Another method for collecting sweat involves the Macroduct system, which uses a coil placed on the skin [23]. The coil is connected to a collector tube to gather the sweat. The variety of these methods illustrates their importance in various scientific contexts and potential applications. These sweat collection methods have the advantage of collecting large amounts of sweat. However, it takes a lot of time or exercise to collect a large amount of sweat. In this study, the use of highly sensitive analytical methods made it possible to analyze much smaller quantities of sweat (<1 μL), and sweat can be collected more easily by using a trace amount of sweat that is constantly generated from the human body.

In this study, amino acid quantification was performed on small sweat samples using a highly sensitive analytical method: high-performance liquid chromatography with fluorescence detection.

## 2 Materials and methods

### 2.1 Chemicals

4-Fluoro-7-nitro-2,1,3-benzoxadiazole (NBD-F) was procured from Dojindo Laboratories (Kumamoto, Japan). The amino acid mixture standard solution Type H and acetonitrile (HPLC grade) were obtained from FUJIFILM Wako Pure Chemical (Osaka, Japan). Purified water was prepared using a Millipore ultra-pure water system (Merck Millipore, Darmstadt, Germany).

### 2.2 Human sweat samples

All studies involving human subjects were approved by the ethical review committees of the Graduate School of Pharmaceutical Sciences, University of Tokyo, Tokyo, Japan (4-2). Sweat samples of four healthy volunteers (male, 21–24 years old) from whom informed consent was obtained were collected in a temperature-controlled room set to 26 °C, although ambient humidity was not regulated. Hand hygiene was maintained by washing hands with running water for 1 min and gently wiping them with KimWipes (Nippon Paper Crecia, Tokyo, Japan). Hands were then air-dried for 10 min without touching any surfaces to avoid contamination. To collect the samples, 2 mL of 10 % ethanol were poured into an open round dish (inner diameter: 35 mm, depth: 10 mm; Tissue Culture Dish, Becton, Dickinson and Company, Franklin Lakes, NJ, USA). The dish was placed over the participant's right palm, rotated to coat the palm with 10 % ethanol, and then gently rocked for 30 s to ensure a thorough collection of substances from the skin surface. The solution in the dish was retained, and the samples were stored at −80 °C until further analysis.

### 2.3 Estimation of the amount of palm sweat volume

The amount of sweat produced by the left palm was measured while sweat was simultaneously collected from the right palm, using a precision-engineered perspiration meter (Pico-Device, Nagoya, Japan). Sweat production was monitored at 1-min intervals for 5 min, and the average of these readings was used as the representative sweat volume. The use of this advanced perspiration meter ensures precise and accurate measurements for the analysis of bilateral palm perspiration.

### 2.4 Fluorescence derivatization of amino acids

A 20 μL aliquot of the amino acid solution was mixed with 40 μL of 20 mM NBD-F in acetonitrile, and then 140 μL of 0.2 M borate buffer (pH 9.0) was added. The mixture was heated in a water bath at 60 °C for 5 min. Afterward, the solution was cooled in an ice bath, and 200 μL of 0.1 M HCl was added to stop the reaction.

### 2.5 HPLC system

The HPLC system comprised a PU-2080 pump (Jasco, Tokyo, Japan), an AS-950 autosampler (Jasco), an Inertsil ODS-4V column (250 × 3.0 mm I.D., 5 μm, GL Sciences, Tokyo, Japan) in a CO-965 column oven (Jasco), and a FP-2020 fluorescence detector (Jasco). The mobile phases used were (A) 10 mM citrate buffer (pH 6.2) containing 75 mM sodium perchlorate and (B) a water-acetonitrile mixture (50/50, v/v). The gradient elution was as follows: 0 min, 10 % B; 20 min, 50 % B; 33 min, 100 % B; 38 min, 100 % B; and 38.1 min, 10 % B. The flow rate was kept at 0.6 mL/min, the column temperature was set at 40 °C, the detection wavelength was 530 nm, and the excitation wavelength was 470 nm.

### 2.6 Method validation

Samples containing 0.01, 0.05, 1, 5, or 10 μM of each amino acid were injected into the HPLC system after derivatization. Calibration curves were generated by plotting the ratio of the peak area of each sample to that of the internal standard (ε-amino-n-caproic acid) against the concentration of these compounds. Least-squares regression was used to calculate the slope, intercept, and correlation coefficient. The limits of quantitation (LOQ) was defined as the sample concentrations that produced signal-to-noise ratios of 10 (S/N = 10). Intra-day and inter-day variations were calculated by repeated analysis (*n* = 4) of sweat samples. The accuracy was confirmed by adding a standard solution of three concentrations to the sweat sample in the linearity range. The spiked quantities of amino acids were 0.05 μM (low), 0.5 μM (middle), and 5 μM (high).

## 3 Results and discussions

### 3.1 Sweat collection methods

In this study, the use of highly sensitive analytical methods made it possible to analyze much smaller quantities of sweat (<1 μL). The challenge is that the exact amount of sweat cannot be precisely determined. When large amounts of sweat are collected, the solution volume can be measured easily, but with small, invisible quantities, it's not possible to determine the exact volume. To address this, a sweat meter was used to measure sweat volume during collection. Sweat was collected from the right palm, while sweat volume was simultaneously measured from the left palm. The study examined perspiration from both palms and found that the sweat production rate from the left palm (2.66 ± 0.07 mg/min) was nearly identical to that from the right palm (2.64 ± 0.06 mg/min). This confirmed that both palms produced similar amounts of perspiration.

### 3.2 Amino acid standards analysis

To analyze very small sweat samples, highly sensitive analytical methods are required. Amino acids are often derivatized before detection, typically using methods like photometry or fluorimetry, since most amino acids do not absorb UV–visible radiation [13,14]. Common derivatization reagents include o-phthaldialdehyde (OPA), phenylisothiocyanate (PITC), 2,3-naphthalene-dicarboxyaldehyde (NDA), and ninhydrin, which are used to improve sensitivity and selectivity. However, these reagents can have limitations, such as instability and insufficient sensitivity. Therefore, the fluorescence derivatization reagent NBD-F was used because it is known to enable highly sensitive detection down to the femtomole (fmol) range.

**Fig. 1:**
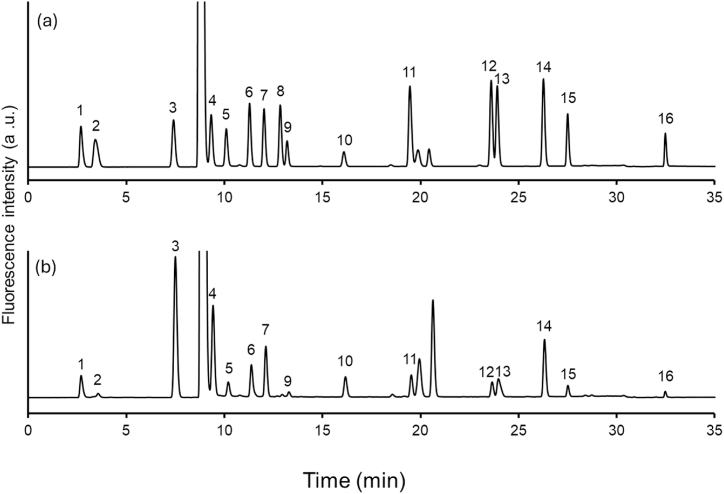
Chromatogram showing separation of fifteen NBD-derivatized amino acids. (a) Amino acid standards and (b) human sweat samples. Peaks: 1, Asp; 2, Glu; 3, Ser; 4, Gly; 5, His; 6, Thr; 7, Ala; 8, Arg; 9, Pro; 10, internal standard (ε-amino-n-caproic acid); 11, Val, 12, Ile, 13, Leu, 14, Phe, 15, Lys, 16, Tyr.

The chromatogram of the NBD-amino acid standard is shown in Fig. 1(a). Fourteen amino acids (Asp, Glu, Ser, Gly, His, Thr, Ala, Pro, Val, Ile, Leu, Phe, Lys, Tyr) were successfully separated within 35 min.

### 3.3 Validation of the developed method

The analytical performance of the developed method is summarized in Table 1, highlighting its linearity. The calibration curve exhibited a high coefficient of determination, greater than 0.9991, across a concentration range of 0.01 (0.05 for Lys) to 10 μM. This calibration curve also allowed for the determination of the limit of quantification (LOQ), resulting in values of 10 nM (50 nM for Lys) as it had a S/N greater than 10 for all analytes. To evaluate the robustness of the assay, the intra-day and inter-day precision were assessed by analyzing four replicates (*n* = 4) of each amino acid.

**Table 1:** Analytical performance of the developed method.

Amino acid	Calibration curve		Intraday precision (%) (*n* = 4)			Interday precision (%) (*n* = 4)		
	Calibration range (μM)	r^2^	Low	Middle	High	Low	Middle	High
Asp	0.01–10	0.9999	2.4	1.1	1.3	2.7	3.1	1.2
Glu	0.01–10	0.9997	2.6	2.1	0.7	2.7	2.5	1.1
Ser	0.01–10	0.9995	0.9	1.7	0.3	1.6	2.1	1.0
Gly	0.01–10	0.9994	2.5	2.6	0.3	1.9	2.1	1.3
His	0.01–10	0.9993	6.9	2.1	0.1	2.5	1.8	1.5
Thr	0.01–10	0.9996	4.6	1.8	0.1	2.8	1.9	1.0
Ala	0.01–10	0.9998	4.4	1.9	0.1	1.8	1.9	0.5
Pro	0.01–10	0.9994	7.6	1.7	2.0	1.9	1.8	1.1
Val	0.01–10	0.9996	4.4	1.9	0.1	2.4	1.8	0.7
Ile	0.01–10	0.9997	1.8	1.6	0.1	3.2	1.8	0.9
Leu	0.01–10	0.9998	4.6	1.9	0.1	3.6	1.8	2.1
Phe	0.01–10	0.9995	2.7	2.1	0.2	2.1	1.9	0.8
Lys	0.05–10	0.9995	2.8	4.3	1.2	4.3	0.6	0.6
Tyr	0.01–10	0.9991	2.7	2.8	0.1	2.8	1.8	0.8
								

Table 2 presents the precision and accuracy of the sweat samples. In terms of precision, the relative standard deviation (RSD) values range from 1.6 % to 10.1 % (n = 4). Accuracy was tested by adding three concentrations of standard amino acids to the sweat samples, with results falling within the acceptable range of 88 %–107 %, demonstrating the assay's reliability across different concentration levels. The precision, with RSD values below 10.1 %, indicates consistent and reproducible results over time. These findings emphasize the assay's reliability and suitability for precise quantification of amino acids across various concentrations, supporting the robustness of the analytical method.

**Table 2:** Precision and accuracy of amino acid measurements in sweat samples.

Amino acid	Precision (RSD, %)	Accuracy (%)		
		Low	Middle	High
Asp	7.2	101	104	105
Glu	1.6	107	105	107
Ser	8.9	88	99	98
Gly	10.1	90	98	94
His	4.0	90	96	98
Thr	7.7	91	99	99
Ala	5.4	92	100	95
Pro	1.6	90	93	92
Val	3.0	93	98	95
Ile	2.3	92	97	95
Leu	2.8	93	96	95
Phe	6.0	92	98	97
Lys	6.6	95	102	98
Tyr	5.1	91	95	94

### 3.4 Amino acid analysis in sweat samples

Fourteen amino acids were detected and quantified in sweat samples (Fig. 1(b)), and the results from 4 healthy volunteers are summarized in Table 3. Amino acid concentrations were calculated based on the volume of sweat. Serine (Ser) was the most abundant amino acid in sweat, followed closely by glycine (Gly) and alanine (Ala). This pattern aligns with previous studies [[19], [20], [21]], lending credibility to the findings.

**Table 3:** Amino acid concentrations in sweat samples.

Asp	214 ± 16.8	Pro	109 ± 7.4
Glu	39.0 ± 4.8	Val	141 ± 7.6
Ser	1664 ± 128	Ile	91.6 ± 13.8
Gly	1024 ± 122	Leu	112 ± 7.2
His	220 ± 17.4	Phe	203 ± 14.4
Thr	287 ± 19.6	Lys	106 ± 8.6
Ala	458 ± 8.0	Tyr	112 ± 23

(mean ± SD, μM, n = 4).

The amino acid composition in sweat samples notably differed from the corresponding profiles in plasma samples. These distinct variations highlight the unique molecular signature of amino acids in sweat, emphasizing the potential of sweat analysis as a distinct and informative avenue for exploring biomarkers. Plasma samples were not collected from volunteers who provided sweat samples. Instead, representative amino acid concentrations previously established in plasma were used as reference data for the analytical framework of this study [24]. This approach ensures ethical considerations while also utilizing established plasma datasets to inform and contextualize the observed amino acid composition in sweat.

Amino acids in sweat originate from the eccrine sweat glands, and their concentrations are influenced by many factors, including hydration status, physical activity, and specific skin conditions. The interaction of these variables gives sweat amino acids a complex and variable profile. This intricate array of influences suggests that sweat amino acids could offer a more direct reflection of local skin metabolism, painting a dynamic picture of the molecular landscape influenced by the microenvironment of the eccrine sweat glands. The noticeable differences in sweat amino acid profiles highlight the unique and complex molecular signatures inherent to this biofluid. The distinctive composition of sweat not only underscores its potential as a valuable medium for exploring biomarkers but also makes it a promising source for revealing physiological nuances, providing a pathway to a deeper understanding of local metabolic processes and their broader systemic implications.

## 4 Conclusions

We quantified the amino acid concentrations in human sweat samples and successfully identified fourteen amino acids with robust validation data. In this study, we were able to quantify amino acids from very small sweat samples taken at rest. To ensure precise quantification, the sweat volume was measured with a sweat meter. This technique has potential for non-invasive and straightforward disease screening, as it allows for the analysis of amino acids in sweat without the need for excessive sweating, like during exercise. It also has potential applications in detecting other biomolecules in sweat besides amino acids.

## Ethical statement

The ethics approval number was 4-2 approved by the ethical review committees of the Graduate School of Pharmaceutical Sciences, University of Tokyo, Tokyo, Japan, and confirmation that informed consent was obtained.

## Funding

This research did not receive any specific funding.

## Data availability statement

No.

## CRediT authorship contribution statement

**Makoto Tsunoda:** Writing – review & editing, Writing – original draft, Methodology, Investigation, Conceptualization. **Takao Tsuda:** Writing – review & editing, Conceptualization.

## Declaration of competing interest

The authors declare that they have no known competing financial interests or personal relationships that could have appeared to influence the work reported in this paper.
